# The effect of justice sensitivity on malevolent creativity: the mediating role of anger and the moderating role of emotion regulation strategies

**DOI:** 10.1186/s40359-024-01759-w

**Published:** 2024-05-13

**Authors:** Yan Wang, Keke Zhang, Fangfang Xu, Yayi Zong, Lujia Chen, Wenfu Li

**Affiliations:** School of Mental Health, Jining Medical University, Jining, 272067 China

**Keywords:** Justice sensitivity, Malevolent creativity, Anger, Emotion regulation

## Abstract

**Background:**

the AMORAL model emphasizes the close connection of individuals’ belief system and malevolent creativity. Belief in a just world theory (BJW) states that people have a basic need to believe that the world they live in is just, and everyone gets what they deserve. Therefore, justice matters to all people. Justice sensitivity, as one of individual trait, has been found associated with negative goals. However, relevant studies have not tested whether justice sensitivity can affect malevolent creativity and its psychological mechanisms. Additionally, researchers have found that both anger and emotion regulation linked with justice sensitivity and malevolent creativity, but their contribution to the relationship between justice sensitivity and malevolent creativity remained unclear. The current study aims to explore the influence of justice sensitivity on malevolent creativity, the mediating effect of trait anger/state anger on the relationship between justice sensitivity and malevolent creativity, and the moderating effect of emotion regulation on this mediating effect.

**Methods:**

A moderated mediating model was constructed to test the relationship between justice sensitivity and malevolent creativity. A sample of 395 Chinese college students were enrolled to complete the questionnaire survey.

**Results:**

Justice sensitivity positively correlated with malevolent creativity, both trait anger and state anger partly mediated the connection between justice sensitivity and malevolent creativity. Moreover, emotion regulation moderated the indirect effect of the mediation model. The indirect effect of justice sensitivity on malevolent creativity through trait anger/state anger increased as the level of emotion regulation increased. The results indicated that justice sensitivity can affect malevolent creativity directly and indirectly through the anger. The level of emotion regulation differentiated the indirect paths of justice sensitivity on malevolent creativity.

**Conclusions:**

Justice sensitivity and malevolent creativity was mediated by trait anger/state anger. The higher sensitivity to justice, the higher level of trait anger/state anger, which in turn boosted the tendency of malevolent creativity. This indirect connection was moderated by emotion regulation, individuals with high emotion regulation are better able to buffer anger from justice sensitivity.

## Background

Creativity is generally believed that it is beneficial and positive. Researchers pointed out that creativity refers to the ability to generate novel and appropriate ideas or products in a specific environment [1]. However, creativity is not always positive. People also have some novel but negative ideas and behaviors—malevolent creativity, which refers to original and premeditated ideation deliberately performed in order to realize one’s own goals and desires, and it always leads to negative consequences, such as new types of fraud, murder, etc [2]. A wide variety of malevolent creativity instances can be found everywhere and cause damage in original or innovative ways, and it is hard to detect and prevent [3]. Therefore, it is of great social significance to reveal the influence factors of malevolent creativity and explore the effective regulation strategies to reduce the potential harm.

The AMORAL model emphasized individuals’ belief system, or their interconnected set of beliefs helped determine whether and to what extent they engage in malevolent creativity. Moreover, the drivers of malevolent creativity also included the need to align actions with belief systems [4]. At the same time, belief in a just world theory (BJW) stated that people had a basic need to believe that the world they lived in is just, and everyone got what they deserved [5, 6]. Researchers had found that individuals were more frequently exhibit malevolent creativity in hostile, angry, injustice, and vengeful situation [7]. Meanwhile, social exchange theory stated that justice was the basis of social exchange and an essential element of effective social interaction. Injustice in an organization or group was a source of stress for its members which were provoked into negative emotions and even outright antisocial aggression by differential treatment [8]. Therefore, the feeling of injustice may matter to malevolent creativity.

A variety of studies examining the distributive, interpersonal, and procedural justice showed that it was perceived justice, not objective circumstances, shaped responses to injustice [9]. Justice sensitivity is an individual trait, which is reflected in the difficulty of detecting injustices and the intensity of the response to injustices. Individuals with high justice sensitivity are more likely to perceive injustice than those with low justice sensitivity [10]. Schmitt et al. categorized justice sensitivity into four types: victim sensitivity, observer sensitivity, beneficiary sensitivity and perpetrator sensitivity [11]. Mohiyeddini and Schmitt found that justice sensitivity performed better than other variables (e.g., trait anger, anger out, and self-assertiveness) in predicting reactions to unfair treatment [12]. There was a study found that individuals tend to establish negative goals when they encountered unfair situations, which may lead to the emergence of malevolent creativity [13]. Another studies also showed that justice sensitivity closely positively correlated kinds of externalizing problems, such as relational, proactive, and reactive aggression in adults [14] and peer victimization [15]. What’s more, Gollwitzer et al. found victim sensitivity was associated negatively with prosocial behavior and positively with antisocial behavior [16]. Prior studies verified that people who have encountered an injustice situation would show more malevolent creativity. However, it remains unknown whether justice sensitivity can affect malevolent creativity and how it affects malevolent creativity. Therefore, the current study focused on the influence of justice sensitivity on malevolent creativity and explored the underlying mechanisms.

Anger is a basic emotional state, according to State-Trait Anger theory, which can be divided into state anger and trait anger [17]. State anger is a temporary emotional state which composed of subjective feelings and physiological activities. On the other hand, trait anger is defined as a stable personality characteristic, a general tendency of angry reaction under the induced stimulus, and a relatively stable individual difference in frequency, intensity and duration of state anger [18]. High-trait angry individuals are more inflamed and easily develop state anger, then show more maladaptive cope including verbal and physical confrontation [19, 20].

Equity theory stated that negative emotions such as anger and resentment were aroused when individuals realized they had been treated unfairly [21]. Social psychological researches indicated that anger was the predominant emotional response to perceiving injustice [22, 23]. A number of empirical studies also examined the relationship between anger and injustice, and indicated that the level of anger was higher when individuals perceived injustice or had been treated unfairly [24–27]. Furthermore, researches showed that facets of anger (i.e., state, trait, expression, inhibition) linked with perceived injustice [28, 29]. Schmitt et al. also found justice sensitivity related with trait anger [30]. Additionally, individuals with high justice sensitivity may be more likely to have a stronger reaction when they accounted injustice events, which might in turn produce a higher degree of state anger.

It has been shown that feeling unfair treatment can give people a sense of relative deprivation [31], which lead to anger and criminal behavior [10, 32, 33]. Anger was an emotion with high arousal and approach orientation which could reinforce cognitive activation state, and allowed the person to mobilize more adequate cognitive resources to engage in the current cognitive activity (e.g., creative thinking). Therefore, anger could facilitate creative performance [34–37]. Cheng et al. conducted an experimental study with the malevolent creativity task (MCT) and found that malevolent creativity performance can be significantly promoted in anger group [38]. Therefore, anger may be a potential mediating variable between justice sensitivity and malevolent creativity.

Previous studies explored induced anger emotion in the laboratory, but few researches examined the relationship between anger and malevolent creativity under natural conditions. There was a study shown that trait anger could significantly and positively predicted aggression [39]. And other study also found that state anger could influence an individual’s tendency to aggression through anger rumination [40]. Thus, the current study speculated that both trait anger and state anger may influence the tendency to malevolent creativity. Additionally, whether trait anger and state anger play a different role between justice sensitivity and malevolent creativity is still unknown. Therefore, both state anger and trait anger deserve attention. Considering the differences between the two kinds of anger, the current study separately examined their roles between justice sensitivity and malevolent creativity.

However, in realistic situations, justice-sensitive individuals do not always produce extreme anger emotion and generate tendency to malevolent creative behavior when they faced with injustice events. This may closely rely on the regulation and control of emotion production, perception, and expression.

Emotion regulation, composed of cognitive reappraisal and expressive suppression, is defined as a series of cognitive processes adjusting or changing the appearance, intensity and duration of emotion [41]. Cognitive behavioural therapy (CBT) approaches proved that angry emotion can be best downregulated by those emotion regulation strategies, such as modifying negative thoughts, or reappraising the anger-provoking situation [42]. Furthermore, according to Gross’s process model of emotion regulation, strategies that act early in the emotion-generative process might differ from the later one in consequences [43]. Cognitive reappraisal is an antecedent-focused strategy used before an emotion occurs, individual can change the emotional experience by altering the perception of a negative event. Expressive suppression, on the other hand, refers to an individual’s ability to alter the external manifestation of emotion by inhibiting expression. That is to say, both can work in the early stages of emotion production. Researchers examined the effects of emotion regulation strategies on both trait anger and state anger, andresults showed that both cognitive reappraisal and emotion suppression can counteract short-term anger arousal following provocation [42]. Numerous studies showed that high level emotion regulation could effectively down-regulate an individual’s anger mood and the related physiological responses [42, 44–46]. Cheng et al. also found that cognitive reappraisal and expressive suppression could effectively reduce the emotional arousal and significantly reduce the malevolent creativity of angry individuals [38]. Based on previous findings, it is reasonable to expect that cognitive reappraisal and expressive suppression may also attenuate the possible effects of justice sensitivity on anger and then weak the impact on malevolent creativity. Therefore, the current study hypothesizes that emotion regulation can play a moderating role between justice sensitivity and anger.

**Fig. 1:**
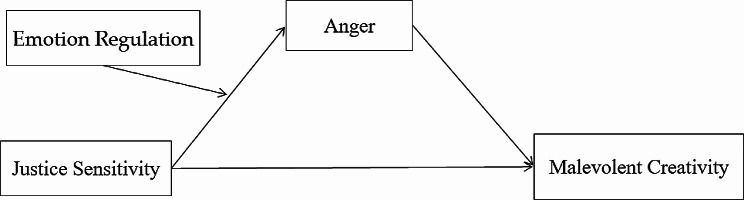
The hypothetical moderated mediation model

In conclusion, the aims of the present study were: (1) to reveal the influence of justice sensitivity on malevolent creativity; (2) to investigate whether trait anger as well as state anger played mediating role in the association between justice sensitivity and malevolent creativity; (3) to explore whether emotion regulation moderated the correlation between justice sensitivity and anger. The hypothetical moderated mediation model was shown in Fig. 1.

## Methods

### Participants

Prior to the beginning of the study, we used the G*Power 3.1. provided by Faul et al. to estimate the required sample size [47]. With setting the medium effect size *f*^2^ = 0.15, α = 0.05, 95% power (1-β err probability), and the number of predictors = 7, the total sample size was 153.

505 college students participated in this study and volunteered for an online survey on the website. A total of 395 valid questionnaires were collected for the study, out of those, 229 (58%) were from male students and 166 (42%) from female students. The major of participants included science and engineering (23%), medicine (25%), literature and history (22%), arts and sports (22%), and others (15%). 347 (88%) were undergraduates, 25 (6%) were postgraduates and 23 (6%) were others.

### Measures

#### Justice sensitivity inventory (JSI)

The Justice Sensitivity Inventory developed by Schmitt was used to measure justice sensitivity [11, 48, 49]. Previous studies had shown that the scale had good reliability and was widely used [10, 50]. This scale consisted of four subscales: victim sensitivity, observer sensitivity, beneficiary sensitivity, and perpetrator sensitivity. Each subscale consisted of 10 questions and was scored on a 6-point Likert scale (e.g. I cannot easily bear it when others profit unilaterally from me.). This scale was scored from strongly disagree to strongly agree as 1–6. The JSI score is the sum of all the item scores. Higher scores indicated higher justice sensitivity. Cronbach’s α for justice sensitivity in this study was 0.97.

#### Trait anger scale (TAS)

Trait anger was measured by the Chinese version of the Trait Anger Scale [20, 51], which consisted of 10 items (e.g. I’m easily irritated.). Studies showed that the scale had good reliability and validity, and widely used in China [52, 53]. This scale was scored on a 4-point Likert scale, and the higher total score indicated higher levels of trait anger. The Cronbach’s α for this scale in this study was 0.87.

#### State anger scale (SAS)

State Anger Scale was developed by Spielberger and revised into Chinese version by Liu [54–56]. The scale had been widely used in China [57]. This scale consisted of 15 items (e.g. I’m angry.) and included three subscales: anger feelings, anger words, and anger actions. This scale was scored on a 4-point scale, with 1 (not at all), 2 (a little), 3 (moderately), and 4 (very strongly). The higher score, the more pronounced state anger. Cronbach’s α for this scale in this study was 0.92.

#### Emotion regulation questionnaire (ERQ)

Emotion regulation was evaluated by a 10-item self-report version of the Emotion Regulation Questionnaire (ERQ). The scale was developed by Gross and revised into Chinese version by Wang et al. [58, 59]. The Chinese version of ERQ had good construct validity, retest reliability, and internal consistency reliability [60, 61]. ERQ was consisted of two subscales, including 6 items (e.g. I control my emotions by changing the way I think about the situation I’m in.) for cognitive reappraisal and 4 items for expressive suppression (e.g. I don’t show my emotions.). This scale was rated on a 7-point. The higher total score indicated more frequent use of emotion regulation strategies. Cronbach’s α for this scale in this study was 0.72.

#### Malevolent creativity behavior scale (MCBS)

Malevolent creativity was measured by MCBS, which developed by Hao et al. and could be used to measure the tendency of individuals to exhibit malevolent creativity behaviors in their daily lives [62]. The scale had a good ecological validity, covered various forms of malevolent creativity (e.g., deception, tricks, lies), and was easy to administer [62]. This scale consisted of 13 items and was scored on a 5-point scale, with 1 (not at all) ∼ 5 (always) (e.g., When I am treated unfairly, I will retaliate in a different way). The scores of all items were summed to obtain the total score. The higher total score indicated that the individual showed more malevolent creativity in daily life. Cronbach’s α for this scale in this study was 0.92.

### Analysis

The descriptive statistical analysis and correlation analysis were conducted using SPSS 26.0. Regression analyses were used to test the mediating role of trait anger / state anger between justice sensitivity and malevolent creativity. PROCESS 3.3 was used to test the moderating role of emotion regulation. The demographic variables (gender, major and grade) were entered in the model as covariates.

## Results

### Common method bias assessment

Harman’s single-factor test was used for exploring the common method bias of the data. All of items of JSI, TAS, SAS, ERQ and MCBS were put into the un-rotated exploratory factor analysis. The results showed that the number of factors with an eigenvalue greater than 1.00 was 17, and the explained variance of the first factor was 28.85, which was lower than the critical criterion of 40% [63]. The results indicated that there was no obvious common method bias in the data of this study.

### Descriptive statistical and correlational analysis

As shown in Table 1, all of the variables were significantly correlated with each other. The score of JSI was significantly positively correlated with the score of TAS, SAS and MCBS, and were significantly negatively correlated with the score of ERQ. Both TAS and SAS were significantly negatively correlated with ERQ and positively correlated with MCBS.

**Table 1:** Descriptive statistics and correlations among study variables (*n* = 395)

	*M* ± *SD*	1	2	3	4	5
1 JSI	113.72 ± 38.77	1				
2 TAS	19.36 ± 6.28	0.379^***^	1			
3 SAS	28.56 ± 9.70	0.313^***^	0.830^***^	1		
4 ERQ	42.67 ± 9.00	-0.232^***^	-0.161^***^	-0.185^***^	1	
5 MCBS	31.46 ± 11.09	0.358^***^	0.548^***^	0.570^***^	-0.161^***^	1

Note: ^***^p < 0.001

### Analysis of the mediating role of trait anger and state anger

Regression analysis was used to test the mediating role of trait anger and state anger between justice sensitivity and malevolent creativity [64, 65].

#### Trait anger as the mediator

**Fig. 2:**
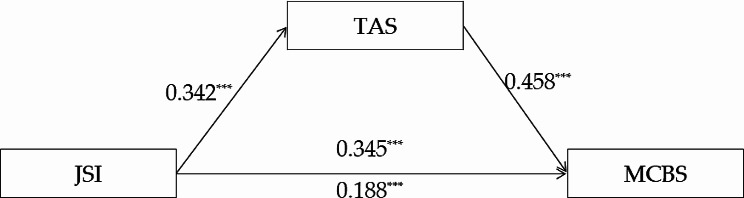
The mediating role of trait anger between justice sensitivity and malevolent creativity

Three regression models were constructed to test the mediating role of trait anger. Firstly, malevolent creativity entered the model as a dependent variable, then demographic variables (gender, major and grade) entered the first block as control variables, and justice sensitivity entered the equation as predictor variable. Secondly, trait anger entered the model as a dependent variable, then demographic variables (gender, major and grade) entered the first block as control variables, and justice sensitivity entered the second block as predictor variable. Finally, malevolent creativity entered the model as a dependent variable, then demographic variables (gender, major and grade) entered the first block as control variables, and justice sensitivity and trait anger entered the equation as predictor variables. The results were shown in Table 2. It showed that justice sensitivity significantly positively predicted malevolent creativity (*c* = 0.345, *t* = 7.262, *p* < 0.001) and trait anger (*a* = 0.342, *t* = 7.392, *p* < 0.001), while trait anger significantly and positively predicted malevolent creativity (*b* = 0.458, *t* = 9.839, *p* < 0.001). Additionally, the direct effect (path *c*’) of justice sensitivity on malevolent creativity was statistically significant (*c*’ = 0.188, *p* < 0.001). Therefore, the mediation model was confirmed, which indicated that the relationship between justice sensitivity and malevolent creativity was partially mediated by trait anger. The indirect effect was 0.157 (95% CI [0.111, 0.209]), which accounted for 45.51% of the total effect. The model diagram was shown in Fig. 2.

**Table 2:** The mediating role of trait anger between justice sensitivity and malevolent creativity

Dependent variable	Independent variable	∆*R*^2^	*R* ^2^	*F*	*β*	*t*	*p*
MCBS	gender	0.059^***^	0.059	8.237	-0.076	-1.547	0.123
	major				0.003	0.067	0.947
	grade				0.229^***^	4.654	0.000
	JSI	0.112^***^	0.171	20.180	0.345^***^	7.262	0.000
TAS	gender	0.103^**^	0.103	14.976	-0.128^**^	-2.680	0.008
	major				-0.062	-1.302	0.194
	grade				0.286^***^	5.956	0.000
	JSI	0.110^***^	0.213	26.434	0.342^***^	7.392	0.000
MCBS	gender	0.059^***^	0.059	8.237	-0.076	-1.547	0.123
	major				0.003	0.067	0.947
	grade				0.229^***^	4.654	0.000
	JSI	0.277^***^	0.377	39.470	0.188^***^	4.142	0.000
	TAS				0.458^***^	9.839	0.000

Note: ^***^p < 0.001; ^**^p < 0.01

#### State anger as the mediator

**Fig. 3:**
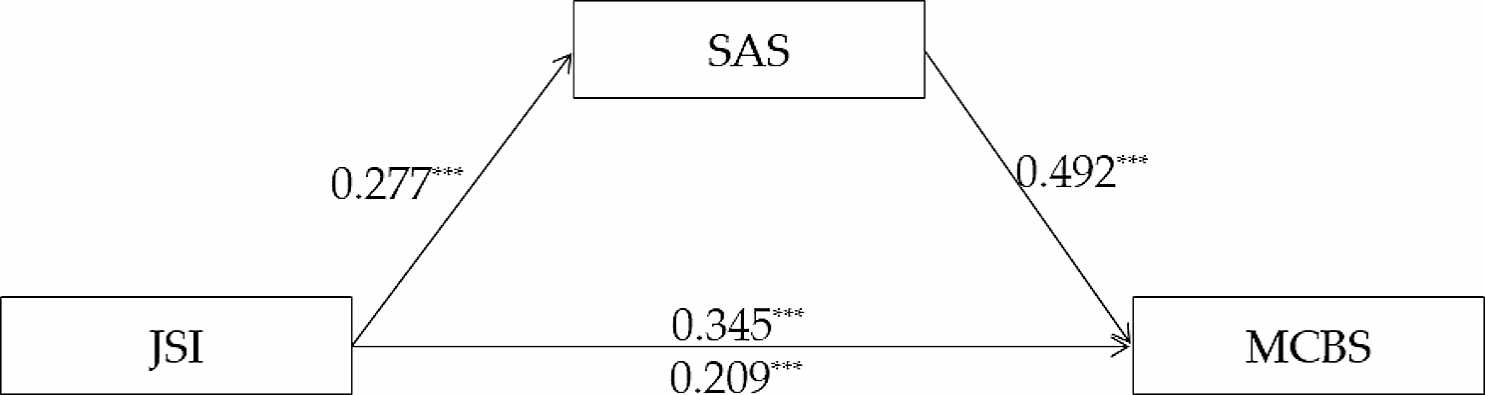
The mediating role of state anger between justice sensitivity and malevolent creativity

Similar to trait anger, hierarchical regression analysis was used to examine the mediating role of state anger. The results were shown in Table 3. It showed that justice sensitivity significantly and positively predicted malevolent creativity (*c* = 0.345, *t* = 7.262, *p* < 0.001) and state anger (*a* = 0.277, *t* = 5.903, *p* < 0.001), while state anger significantly and positively predicted malevolent creativity (*b* = 0.492, *t* = 10.958, *p* < 0.001). Therefore, the mediation model was confirmed, which indicated that the relationship between justice sensitivity and malevolent creativity was partially mediated by state anger. The indirect effect was 0.136 (95% CI [0.087, 0.188]), which accounted for 39.50% of the total effect. The model diagram was shown in Fig. 3.

**Table 3:** The mediating role of state anger between justice sensitivity and malevolent creativity

Dependent variable	Independent variable	∆*R*^2^	*R* ^2^	*F*	*β*	*t*	*p*
MCBS	gender	0.059^***^	0.059	8.237	-0.076	-1.547	0.123
	Major				0.003	0.067	0.947
	grade				0.229^***^	4.654	0.000
	JSI	0.112^***^	0.171	20.180	0.345^***^	7.262	0.000
SAS	gender	0.119^***^	0.119	15.597	-0.165^**^	-3.475	0.001
	major				0.001	0.011	0.992
	grade				0.297^***^	6.237	0.000
	JSI	0.072^***^	0.191	34.843	0.277^***^	5.903	0.000
MCBS	gender	0.059^***^	0.059	8.237	-0.076	-1.547	0.123
	major				0.003	0.067	0.947
	grade				0.229^***^	4.654	0.000
	JSI	0.367^***^	0.377	94.463	0.209^***^	4.811	0.000
	SAS				0.492^***^	10.958	0.000

Note: ^***^p < 0.001, ^**^p < 0.01

### Analysis of the moderating role of emotion regulation

**Fig. 4:**
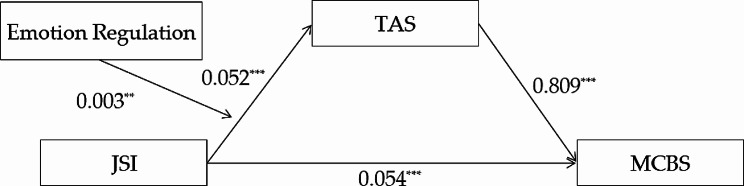
The moderated mediation models (trait anger as the mediator)

**Fig. 5:**
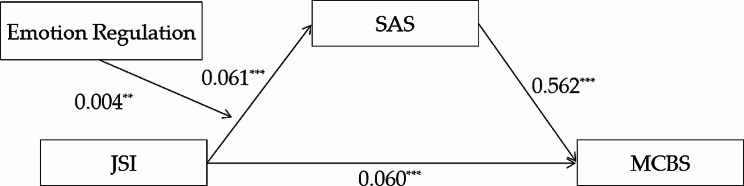
The moderated mediation models (state anger as the mediator)

Model 7 in PROCESS 3.3 developed by Hayes was used to explore the hypothesized moderated mediation model [66], as shown in Figs. 4 and 5, the indirect association between justice sensitivity and malevolent creativity was moderated by emotion regulation. The results showed that the interaction of justice sensitivity and emotion regulation significantly predicted trait anger (B = 0.003, *t* = 3.021, *p* = 0.003), as well as state anger (B = 0.004, *t* = 2.765, *p* = 0.006).

**Fig. 6:**
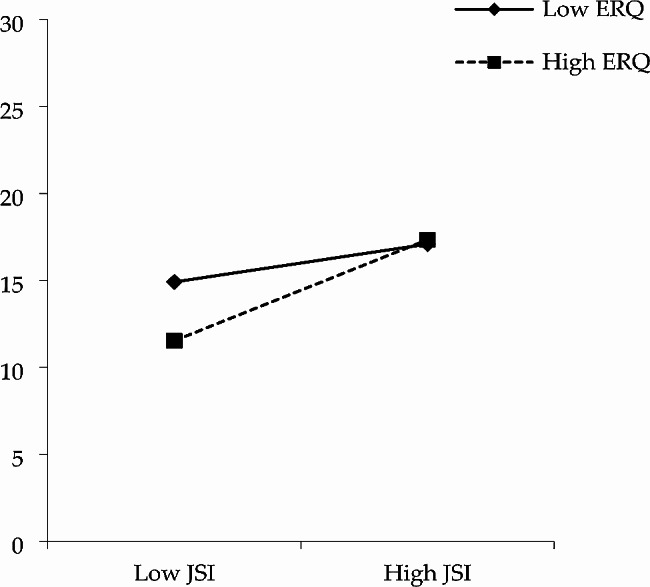
The interaction effect of JSI and ERQ on TAS

**Fig. 7:**
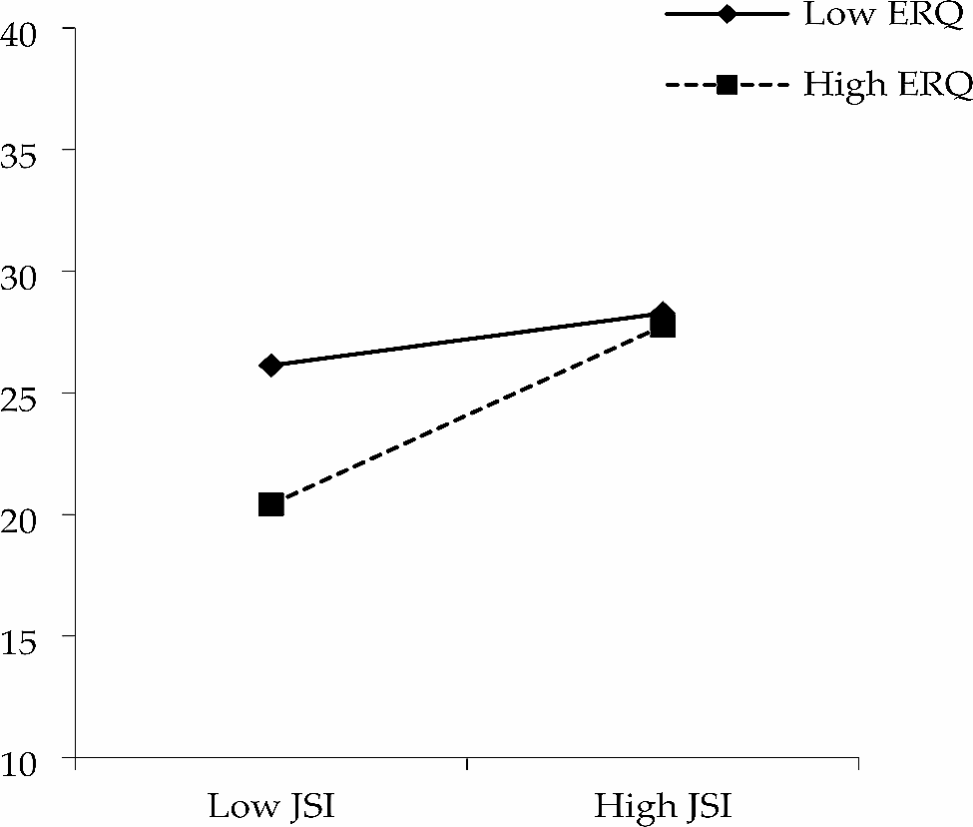
The interaction effect of JSI and ERQ on SAS

As shown in Figs. 6 and 7, justice sensitivity could significantly and positively predict trait anger (*β* = 0.075, *t* = 7.024, *p <* 0.001) and state anger (*β* = 0.095, *t* = 5.532, *p <* 0.001), when the level of emotional regulation was high. Meanwhile, justice sensitivity could significantly predict trait anger positively (*β* = 0.028, *t* = 2.538, *p* = 0.012) instead of state anger (*β* = 0.028, *t* = 1.560, *p* = 0.120), when the level of emotional regulation was low. Additionally, justice sensitivity had a stronger predictive effect on trait anger and state anger when the level of emotion regulation was higher. The results suggested that higher levels of emotion regulation could serve as a buffer against the influences of justice sensitivity on trait anger and state anger among low justice sensitivity individuals. However, the moderating effect of emotion regulation was no longer significant when an individual’s justice sensitivity was high.

The effects of emotion regulation on the mediating pathway of justice sensitivity → trait anger → malevolent creativity (index = 0.0021, SE = 0.0007, 95% CI: [0.0008, 0.0036]) and justice sensitivity $\to$ state anger $\to$ malevolent creativity (index = 0.0021, SE = 0.0009, 95% CI: [0.0005, 0.0039]) were all statistical significant. The details were shown in Table 4. The indirect effects through trait anger were both significant in participants with high and low emotion regulation. Meanwhile, the indirect effects through state anger were significant in participants with high emotion regulation and not those with low emotion regulation.

**Table 4:** The moderated indirect effect

Mediator	Moderator	Effect	SE	95% CI	
TAS	*M* - *SD*	0.0227	0.0090	0.0058	0.0415
	*M*	0.0417	0.0076	0.0278	0.0578
	*M* + *SD*	0.0606	0.0107	0.0411	0.0831
SAS	*M* - *SD*	0.0155	0.0112	-0.0053	0.0384
	*M*	0.0343	0.0079	0.0201	0.0513
	*M* + *SD*	0.0531	0.0113	0.0326	0.0772

## Discussion

To advance the understanding of malevolent creativity, the present study investigated a moderated mediation model to revealed the association between justice sensitivity and malevolent creativity. As hypothesized, the correlation between justice sensitivity and malevolent creativity was mediated by trait anger/state anger. The higher sensitivity to justice, the higher level of trait anger/state anger, which in turn boosted the tendency of malevolent creativity. Additionally, this indirect connection was moderated by emotion regulation. To be specific, the indirect effects through trait anger were both significant in participants with high and low emotion regulation, however, the indirect effects through state anger were significant in participants with high emotion regulation but not those with low emotion regulation.

### The association between justice sensitivity and malevolent creativity

The results of this study found that justice sensitivity significantly positively predicted malevolent creativity, which was in line with prior researches. Individuals who were not treated fairly would experience more negative emotions and show more negative behaviors. For example, Brebels et al. found that participants who faced unequal distributional outcomes stole more money from the manager [67]. Another study found that organizational injustice perception which included procedural justice and interpersonal justice could negatively predict workplace deviance, and the relationships mediated by negative emotion [68].

Justice is an important means to defend self-benefit in society. The Sensitivity to Mean Intentions Model (SeMI) states that individuals with higher level of justice sensitivity have a lower threshold for perceiving malicious information in offensive or threatening situations compared to individuals with low justice sensitivity. That’s why high justice sensitivity individuals tend to actively search for or focus on information unfavorable to them, and then activate a suspicious mindset after perceiving malicious intentions [69]. Therefore, individuals with high justice sensitivity were more attentive to unfair stimuli and activated easily by the unfair information, which might prompt them to take steps to defend the fairness and self-benefit [10]. As a result, these people might tend to engage in more negative deviant behaviors [70], and be more likely to harm others, i.e., show more malevolent creativity. Previous studies demonstrated that people tend to exhibit malevolent creativity in threatened context, e.g. bullying victimization [71], unfair [13]. Clark and James found that perceptions of unfair treatment enhanced instances of negative creativity whereas perceptions of fair treatment yielded more positive creativity [13]. Another research also found individuals who were more implicitly aggressive and less premeditative were more likely to be malevolently creative in response to situations that provoke malevolent creativity [7]. These results might indicate that situational perceptions, such as justice and fairness, could influence the degree to which creative products are negative. Therefore, high level justice sensitive may generate high level malevolent creativity.

In other hand, justice sensitive individuals do not entirely behave in accordance with norms of justice, sometimes they could show protest and retaliate more strongly at once when they counter injustice [69]. For example, researchers found victim-sensitive individuals tended to make unfair offers when they had the power to distribute money at will between themselves and another person [72]. Another study also showed higher victim sensitivity predicted higher relational, proactive, and reactive aggression, and higher observer sensitivity predicted higher physical and verbal aggression [14]. Schmitt et al. found that vengeful reactions of laid-off employees toward their former employer depended directly and indirectly—mediated by the perceived fairness of the lay-off procedure—on justice sensitivity [73]. Meanwhile existing studies found that individuals who tend to break rules or had a weak sense of rule compliance were more likely to possess higher creativity [74].

In summary, it is plausible that justice sensitivity positively predicts the tendency of malevolent creativity. Individuals with high justice sensitivity are more likely to perceive information about injustice and generate aggressive thoughts and behaviors. This may mean that justice sensitivity individuals also have a tendency to break the rules. The aggressive performance may send a message to the perpetrator that what he has done is reprehensible, at the same time, the performance also is a way to respond injustice in order to avoid similar harm in the future [75]. Thus, individuals with higher justice sensitivity are more likely to generate aggressive thoughts or behaviors and harm others intentionally, so that resulting in higher level of malevolent creativity.

### The mediating role of anger

The results of this study found that both trait anger and state anger mediated the relationship between justice sensitivity and malevolent creativity. Specifically, justice sensitivity could not only directly affect malevolent creativity but also indirectly affect malevolent creativity through trait anger/state anger. This result was consistent with previous studies. Some researchers found that justice sensitivity positively predicted anger [38, 76, 77]. Schmitt et al. described justice sensitivity as: “Individuals differ in how sensitive they are to justice; how easily they are able to perceive injustice; and how strongly they react to perceived injustice” [11]. Thus, justice sensitivity was a good predictor of an individual’s response to injustice, those with high justice sensitivity tended to respond more strongly to injustice. To be specific, individuals with high justice sensitivity, when confronted with an injustice allocation scheme, would produce a significant increase in the level of negative emotional arousal, which further lead to an increase in anger [10]. The state of anger, on the other hand, exacerbated the conflict and mistrust in society, undermined the interpersonal interaction and cooperation. Under the emotion of anger, individuals were able to generate more creative and more damaging ideas, which were destructive to society and others [38]. Lastly, this increased the level of malevolent creativity tendency.

### The moderating role of emotion regulation

Our results also revealed that emotion regulation moderated the effect of justice sensitivity on trait anger and state anger. Individuals with high levels of emotion regulation were more likely to avoid anger triggered by justice sensitivity than individuals with low emotion regulation. There is a plausible explanation regarding the moderate role of emotion regulation. As mentioned earlier, individuals who perceive unfairness typically experienced high levels of emotional arousal, while both cognitive reappraisal and expressive suppression emotion regulation strategies were effective in decreasing emotional arousal and implicit aggression [38]. Thus, individuals with high levels of emotion regulation were better able to regulate anger arising from perceived injustice, which in turn reduced the level of malevolent creativity tendency.

Additionally, the current study found that the moderating effect of emotion regulation was different on trait anger and state anger. Justice sensitivity could positively predict trait anger when the level of emotional regulation was low. One possible explanation for this difference is that the lower level of ERQ might suggest that individuals do not need emotion regulation strategies to manage their emotions frequently. This might indicate that people do not receive external injustice information frequently, so that justice sensitivity as a stable personality trait only can predict the trait anger that has a tighter relationship to it [11], instead of state anger. Because state anger always depends on the environmental stimuli in the moment.

Although this study revealed possible mechanisms by exploring justice sensitivity influenced on malevolent creativity, there were still some shortcomings. Firstly, justice sensitivity contained multiple components that were not examined separately in this study. Future research could delve into the relationship between different components of justice sensitivity and malevolent creativity. Secondly, this study did not examine whether there was a difference in the role of cognitive reappraisal and expression suppression, which could be further explored in future studies. Future studies also could choose to incorporate other types of emotion regulation strategies and compare the effects of different emotion regulation strategies. Finally, the MCBS was utilized in current study to measure the level of malevolent creativity. Notably, the MCBS, as a measurement tool, could measure potential propensity of malevolent creativity. Some recent studies related to malevolent creativity used malevolent creativity tasks (MCT) to explore malevolent creativity performance. Future research could combine examination of malevolent creativity propensity and malevolent creativity performance to explore the influencing factors and internal mechanisms of malevolent creativity.

## Conclusions

In conclusion, the present study validated the association between justice sensitivity and malevolent creativity. The findings illustrated the mediating effect of trait anger/state anger in the pathway from justice sensitivity to malevolent creativity. Additionally, the results also showed evidence of two-way interaction, indicating that emotion regulation moderated the relationship between justice sensitivity and anger. Individuals with high emotion regulation are better able to avoid anger from heightened justice sensitivity than individuals with low emotion regulation.

## Data Availability

No datasets were generated or analysed during the current study.

## Abbreviations

JSI: Justice Sensitivity Inventory
TAS: Trait Anger Scale
SAS: State Anger Scale
ERQ: Emotion Regulation Questionnaire
MCBS: Malevolent Creativity Behavior Scale
SD: Standardized deviation
SE: Standard error
95%CI: 95% Bootstrap Confidence interval
